# Effect on lifespan of high yield non-myeloablating transplantation of bone marrow from young to old mice

**DOI:** 10.3389/fgene.2013.00144

**Published:** 2013-08-07

**Authors:** Marina V. Kovina, Viktor A. Zuev, German O. Kagarlitskiy, Yuriy M. Khodarovich

**Affiliations:** ^1^A.N. Bach Institute of BiochemistryMoscow, Russia; ^2^N.F. Gamaleya Institute for Epidemiology and MicrobiologyMoscow, Russia; ^3^M.M. Shemyakin and Y.A. Ovchinnikov Bioorganic Chemistry InstituteMoscow, Russia

**Keywords:** bone marrow transplantation, stem cells, longevity, life extension

## Abstract

Tissue renewal is a well-known phenomenon by which old and dying-off cells of various tissues of the body are replaced by progeny of local or circulating stem cells (SCs). An interesting question is whether donor SCs are capable to prolong the lifespan of an aging organism by tissue renewal. In this work, we investigated the possible use of bone marrow (BM) SC for lifespan extension. To this purpose, chimeric C57BL/6 mice were created by transplanting BM from young 1.5-month-old donors to 21.5-month-old recipients. Transplantation was carried out by means of a recently developed method which allowed to transplant without myeloablation up to 1.5 × 10^8^ cells, that is, about 25% of the total BM cells of the mouse. As a result, the mean survival time, counting from the age of 21.5 months, the start of the experiment, was +3.6 and +5.0 (±0.1) months for the control and experimental groups, respectively, corresponding to a 39 ± 4% increase in the experimental group over the control. In earlier studies on BM transplantation, a considerably smaller quantity of donor cells (5 × 10^6^) was used, about 1% of the total own BM cells. The recipients before transplantation were exposed to a lethal (for control animals) X-ray dose which eliminated the possibility of studying the lifespan extension by this method.

## INTRODUCTION

It is presently held that many tissues of an adult organism are capable of regeneration (self-renewal) by means of resident or circulating stem cells (SCs). The self-renewal of tissues occurs continuously. In the heart of rats, for example, about 7% of the cells are replaced every month (Kajstura et al., 1996), whereas the renewal of blood and epithelial tissues proceeds much faster. The participation of not only local but also circulating SC in the renewal of tissues has been shown by numerous studies on sex-mismatched transplantation which was accompanied by significant y/x-chimerism (Körbling et al., 2002; Herzog et al., 2003; Thiele et al., 2004). The significance of these studies is especially increased now since it has become possible to create cellular material for transplantation that is genetically identical to cells of the patient (Grewal et al., 2004; Takahashi and Yamanaka, 2006; Wang et al., 2011).

Our previous *in vitro* studies have demonstrated that non-differentiated SC can indeed, under certain conditions, be effectively differentiated into the cell type corresponding to their cellular microenvironment (Kovina and Khodarovich, 2011). These findings may explain the previously found, and not enough accounted for, effectiveness of bone marrow transplantation (BMT) in treating not only hematology diseases (Fanconi anemia; Gluckman et al., 1995) but also such systemic diseases as mucopolysaccharidosis and senile hearing impairment (Birkenmeier et al., 1991; Iwai et al., 2001; Corti et al., 2004; Willenbring et al., 2004). Mucopolysaccharidosis is caused by a deficiency in enzymes required for degradation of glucosamines that are accumulated in lysosomes of many organs, thereby leading to dysfunction and reduction of lifespan. Transplantation of syngenic bone marrow (BM) cells from healthy mice resulted in an increase of lifespan from 6 months to the control value of 2 years. The lysosomal activity was recovered in full or in part in all studied tissues. For the thymus gland, spleen, and BM the recovery was complete, for the lungs it was 50%, for kidney and liver, 20%, and for the brain, 7% (Birkenmeier et al., 1991). This result can be logically accounted for by tissue replacement with progeny of donor BM; however, the authors did not determine the degree of chimerism. In the next work of this series, the curing of a hereditary skin disease by BMT was shown; the chimerism of skin was determined to be between 10 and 30%, and the chimerism of the mucous epithelium of the gastrointestinal tract was 50% (Wagner et al., 2010). These facts led to the assumption that the BM, besides hematopoietic and stromal cells, contains cells capable to be differentiated into mature cells of many other tissues (Herzog et al., 2003).

Nevertheless, it is still unclear whether such regeneration can retard the normal process of aging. The BMT methods employed before demanded a strong X-ray irradiation which negatively affected the lifespan of control animals (Birkenmeier et al., 1991). Though there are some life extension reports about myeloablating SC transplantation on mice (Shen et al., 2011), the usage of lethal irradiation cannot be recommended for human anti-aging therapy. In the present work, we report the influence upon lifespan of high yield non-myeloablating transplantation of BM from young mice to old mice.

## MATERIALS AND METHODS

### ISOLATION OF BONE MARROW

All animal experiments were conducted in accordance with the Guiding Principles in the Use of Animals in Toxicology (http://www.toxicology.org/ai/air/air6.asp) and were approved by Institutional Animal Care and Use Committee (IACUC). The donors of BM were C57BL/6 mice aged 6 weeks. The donors were sacrificed by cervical dislocation and sanitized with 70% ethanol. Isolation of BM was carried out as described (Colvin et al., 2004) with small variations. Firstly, the spine and skull were cut out and freed of surrounding tissues using sterile scalpel, scissors, and forceps. The caudal part of the spine was removed. The spine and skull bones were then minced with sterile pistil and mortar in 1–2 ml of cooled sterile Hanks’ balanced salt solution (HBSS) buffer of the following composition: 0.44 mM potassium phosphate, 5.37 mM potassium chloride, 0.34 mM dibasic sodium phosphate, 136.89 mM sodium chloride, 5.55 mM D-glucose. The mixture was filtrated through four layers of a fine-mesh nylon-6 tissue, washed in fresh HBSS buffer and then centrifuged under mild conditions (50 g). The deposit was resuspended in 1 ml HBSS, the cells were counted in a Goryaev chamber and HBSS was added to a final concentration of 5 × 10^7^ cells per ml. The overall number of isolated cells of various sizes and morphology was 3.5–4.5 × 10^8^.

### TRANSPLANTATION OF BONE MARROW

Bone marrow was transplanted to C57BL/6 mice aged 21.5 months. Pre-treatment of suspension of the cells included filtration through four layers of a fine-mesh nylon-6 tissue and addition of 5 U of heparin (Synthesis, Russia) per 2.5 × 10^7^ cells in 0.5 ml HBSS to prevent occlusions of vessels with cellular material. The tail of a mouse was warmed in water at 50–55°C until the twin caudal veins were clearly seen, and then the cell suspension was slowly (during 20 s) injected into one of the veins by means of an insulin syringe.

Transplantations were carried out twice a day on three subsequent days with an interval of 8–16 h, which brought the amount of transplanted cells up to 1.5 × 10^8^ per recipient.

The control animals did not receive any treatment.

### STATISTICAL TREATMENT OF DATA

Approximation of experimental results was done using the Origin software (OriginLab, USA) on the basis of the Gompertz function, traditionally used for survival description (Anisimov et al., 2003; Gavrilov and Gavrilova, 2006):

(1) F(t)=100×exp[−P1×exp(P2×t−P3)],

where *F*(*t*) is the percentage of animals that attained age *t*, 100% is the number of animals at the age 21.5 month (the age-point when the transplantation was performed), and P1, P2, and P3 are parameters to adjust. To find the best fit, we conducted a three-parametric fit for each experimental curve and obtained three pairs of parameters. Then the least sensitive parameter (P1) was averaged and fixed for a two-parametric fit (P2 and P3). Then again the least sensitive (P3) was averaged and fixed for a one-parametric fit (P2).

To find the 50%-survival time (*t*_1/2_) for each curve, we used the following equation, derived from Eq. 1:

(2) $$\begin{array}{c} 50=100\times \exp[-P1\times \exp(P2\times t_{1/2}-P3)], \end{array}\;t_{1/2}=[P3+In((In(2))/P1)]/P2\;\;\;$$

## RESULTS AND DISCUSSION

In the beginning, we selected conditions for massive transplantation of BM cells to mice. According to the idea of the experiment, we needed to inject about 150 million cells (1.5 × 10^8^) to each animal, which amounts to nearly one-fourth of overall BM cells of an adult mouse. It turned out that after injection of a large volume (over 1 ml) or of a highly concentrated cell suspension (1–1.5 × 10^8^/ml) most mice died within 2–10 min. Therefore, the following conditions of transplantation were chosen: the cells were injected in six portions of 2.5 × 10^7^ cells in 0.5 ml at intervals of 8–16 h. An important factor for successful transplantation was the addition of 5 U of heparin per cell dose (2.5 × 10^7^ cells in 0.5 ml) and a thorough filtration of the cells before injection; combining these two measures greatly reduced post-transplantation death of the animals.

**FIGURE 1:**
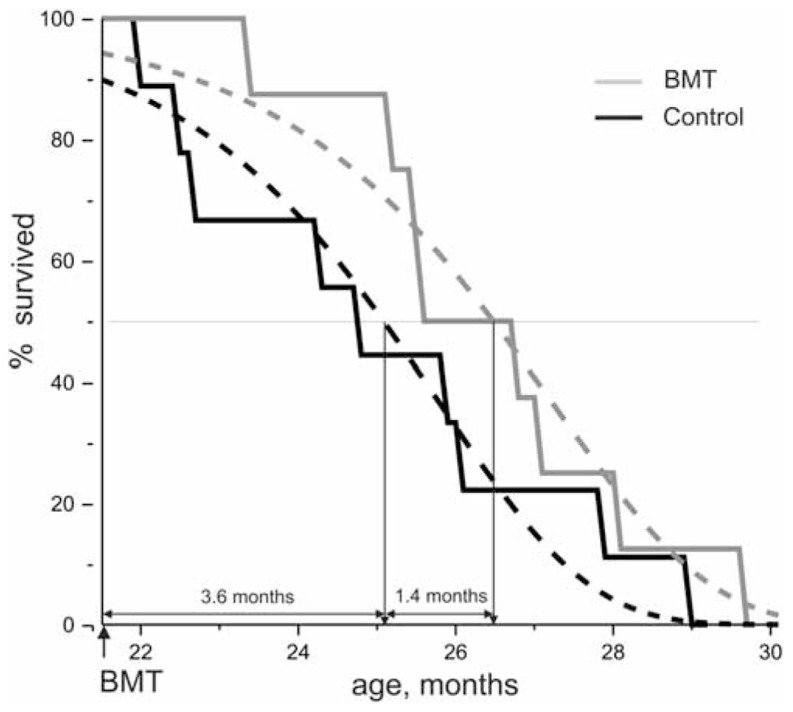
**Effect of massive non-myeloablating syngenic transplantation of bone marrow on the lifespan of C57BL/6 mice**. Black line – Kaplan–Meier graph for animals from the control group (*solid line*) and its Gompertz approximation (*dashed line*); gray line – Kaplan–Meier graph for animals from the experimental group which underwent bone marrow transplantation (*solid line*) and its Gompertz approximation (*dashed line*). BMT, bone marrow transplantation.

The recipients of BM cells were C57BL/6 mice at the age of 21.5 months. Each of the 10 females received 1.5 × 10^8^ cells obtained from 6-week-old males of the same line. In each case the quantity of live BM cells exceeded 90%, which was ascertained by counting cells stained with trypan blue in a Goryaev chamber. Two recipients were lost during transplantations, presumably from formation of thrombi in large vessels; they were excluded from the statistical analysis. The remaining eight mice were observed until their natural death in parallel to nine mice of the same age from a control group not exposed to transplantation. **Figure 1** depicts the effect of BM transplantation on lifespan. The gray line corresponding to the group of recipients is always above the black line of the control group.

Statistical analysis of the results was performed as described in Section “Materials and Methods.” The Gompertz equation describes the experimental data sufficiently good, with the correlation coefficient 0.9. The best fit gave the following values of the mean total lifespan:

$$t_{1/2cntrl}=25.1\pm 0.1\,months\,and\,t_{1/2trnspl}=26.5\pm 0.1\,months.$$

Then, the mean residual lifespan, calculated from the beginning of the observations (age = 21.5 months), was 25.1–21.5 = 3.6 ± 0.1 months and 26.5–21.5 = 5.0 ± 0.1 months for the control and experimental groups, respectively, corresponding to a gain of 5.0–3.6 = 1.4 months (**Figure 1**). Hence, the BMT carried out using the described procedure resulted in a (1.4/3.6) × 100% = 39 ± 4% increase of the mean survival time counting from the moment of transplantation. While the overall lifespan extension is modest (100 × 1.4/25.1 = 6%) we discuss here only the post-transplantation extension (39%), since it properly reflects the effect of the treatment while the overall ratio includes the untreated time. This is a common practice (Dhahbi et al., 2004). The untreated time is several fold greater than post-treatment time and, therefore, cannot be ignored as might be done when the treatment starts soon after weaning. There is no known method to extend the mean wt-mouse life for more than 40% after the start of the treatment, even with dietary restriction, the most powerful method of life extension.

The oldest transplanted animal lived 3 weeks longer than the oldest control animal., However we cannot calculate the maximal lifespan here, since it is, by definition, the mean lifespan of the most long-lived 10% of each group. In our small group, 10% would be less than one mouse. So, the investigation of an influence of BMT on maximal lifespan is the task for future work.

The obtained positive influence of BMT on the mean lifespan in our work is underestimated because of transplantation complications (including the occlusion of vessels) from which, obviously, suffered not only the two mice that died during transplantation and were excluded from the statistics, but also those that survived, though to a lesser degree. We expect a greater difference in lifespan between control and experimental groups by (i) the use of high-quality commercial filters for purification of transplanted material from cell aggregates and (ii) the use of more accurate controls injected with old BM (in this work the control animals did not get the parallel invasive treatment because of the absence of additional 20 months old animals to produce old BM for control transplantation).

Data from the literature also suggest an effect of BMT on the lifespan of mice. For instance, an attempt was made in 2004 to extend the lifespan with syngenic radiation-free transplantation of 4–10 × 10^6^ BM cells from young donors (Kamminga et al., 2005). The effect was not large though (less than 10% of the survival increase), presumably because a relatively small number of cells were transplanted; the resulting chimerism of the BM was also quite modest (1–10%). However, very soon another research team managed to achieve 30–37% chimerism of young recipients after transplantation of 2 × 10^8^ BM cells without irradiation (Colvin et al., 2004), which is in a perfect agreement with the SC competition hypothesis (an adult mouse has a total of 6 × 10^8^ BM cells). The SC competition hypothesis predicts a still greater effectiveness of replacement of the aging recipient’s BM with young donor’s SCs, as the amount of SCs decreases sharply with age.

In perfect agreement with this prediction, the chimerism of old recipients in the above-mentioned study (Kamminga et al., 2005) was 10- to 20-fold greater than chimerism found in younger recipients (5–10 vs. 0.5–1%). Therefore, when the amount of donor cells is increased 10–20 times (from 5–10 × 10^6^ to 1–2 × 10^8^) one may expect an almost complete replacement of the aged BM with that from the donor. Thus, our results which demonstrate a significant effect of BMT on mice survival may be explained as solely by a blood renewal effect (for example, immune boosting effect or increased blood oxygenic capacity), as well as by large number of transplanted cells incorporated in the solid organs and differentiated into the surrounding tissue, thus renewing it. The last conclusion is supported by the results of our previous work on contact differentiation *in vitro*, where we achieved nearly 100% differentiation of SCs into endotheliocytes after seeding them into a primary endothelial culture (Kovina and Khodarovich, 2011).

In our work, we used a slightly different source of BM as it is obtained traditionally – spine and scull instead of femurs. Spine and scull together contain the majority (63%) of total body BM cells, according to Colvin et al. (2004). Besides, BM from spine and scull has a twice higher percentage of high proliferative potential colony-forming cells, than femurs (Colvin et al., 2004). So, it must have a higher potential to renew old tissues than femur’s BM. Finely, spine and scull are easily cleaned from surrounded tissues. Thus, in order to recover the maximal amount of active BM within minimal time, we decided to use only spine and scull as BM sources. A quantitative assessment of the degree of post-transplantation chimerism and its correlations with lifespan on a larger quantity of animals with variations in both: the sources of circulating SC and the lines of recipients will be the next stage of our work. A success along these lines will allow developing approaches for therapy for not only a number of hereditary diseases but also for retardation of the aging process in general.

## Conflict of Interest Statement

The authors declare that the research was conducted in the absence of any commercial or financial relationships that could be construed as a potential conflict of interest.
